# Autophagy Counterbalances Endoplasmic Reticulum Expansion during the Unfolded Protein Response

**DOI:** 10.1371/journal.pbio.0040423

**Published:** 2006-11-28

**Authors:** Sebastián Bernales, Kent L McDonald, Peter Walter

**Affiliations:** 1 Howard Hughes Medical Institute, University of California San Francisco, San Francisco, California, United States of America; 2 Department of Biochemistry and Biophysics, School of Medicine, University of California San Francisco, San Francisco, California, United States of America; 3 Electron Microscope Laboratory, University of California Berkeley, California, United States of America; Massachusetts Institute of Technology, United States of America

## Abstract

The protein folding capacity of the endoplasmic reticulum (ER) is regulated by the unfolded protein response (UPR). The UPR senses unfolded proteins in the ER lumen and transmits that information to the cell nucleus, where it drives a transcriptional program that is tailored to re-establish homeostasis. Using thin section electron microscopy, we found that yeast cells expand their ER volume at least 5-fold under UPR-inducing conditions. Surprisingly, we discovered that ER proliferation is accompanied by the formation of autophagosome-like structures that are densely and selectively packed with membrane stacks derived from the UPR-expanded ER. In analogy to pexophagy and mitophagy, which are autophagic processes that selectively sequester and degrade peroxisomes and mitochondria, the ER-specific autophagic process described utilizes several autophagy genes: they are induced by the UPR and are essential for the survival of cells subjected to severe ER stress. Intriguingly, cell survival does not require vacuolar proteases, indicating that ER sequestration into autophagosome-like structures, rather than their degradation, is the important step. Selective ER sequestration may help cells to maintain a new steady-state level of ER abundance even in the face of continuously accumulating unfolded proteins.

## Abstract 2

The authors describe an ER-specific autophagic process induced by the unfolded protein response (UPR), which sequesters ER into autophagosome-like bodies. This process may be involved in re-establishing homeostasis when unfolded proteins accumulate inside cells.

## Introduction

Secretory proteins and most integral membrane proteins enter the secretory pathway at the endoplasmic reticulum (ER) [1], where they fold and, if appropriate, become covalently modified and assembled into higher order complexes. ER-resident chaperones and other modifying enzymes assist as proteins achieve their active, three-dimensional conformation. Only properly folded and assembled proteins are allowed to leave the ER, thus providing exquisite quality control to ensure fidelity of plasma membrane and secreted proteins through which cells communicate with their environment [2]. This process is regulated at multiple levels to ensure that ER folding capacity is sufficient and adjusted appropriately according to need, i.e., that ER homeostasis is maintained. Cells regulate, for example, the amount of protein translocated into the ER, the concentration of chaperones and other ER enzymes, the abundance of the ER membrane system, and the degradation of unfolded proteins [3–5].

At the center of this regulation is a phylogenetically conserved ER-to-nucleus signaling pathway–called the unfolded protein response (UPR)–that adjusts ER abundance in response to the accumulation of unfolded proteins [6]. Unfolded proteins result when protein folding demand exceeds the protein folding capacity of the ER. The ER-resident transmembrane kinase/endoribonuclease Ire1 is a primary sensor for unfolded proteins in the ER [7–9]. It transmits this information to the cytosol by activating its endoribonuclease domain, which initiates an unconventional mRNA splicing reaction [10–13]. Splicing removes a short intron from a single mRNA species, *HAC1,* allowing the production of an active transcription activator Hac1^i^ [13,14] (or its metazoan ortholog XBP1 [15–17]). Hac1^i^ (or XBP1) then transcriptionally activates a vast set of UPR target genes that in yeast represents more than 5% of the genome [18]. Induction of the UPR target genes increases the biosynthesis of chaperones and modifying enzymes needed to fold proteins, as well as factors involved in transport through the secretory pathway, ER-associated protein degradation (ERAD), and phospholipids biosynthesis. The UPR therefore drives a comprehensive program that adjusts the cell's capacity to fold, process, and secrete proteins.

In metazoan cells, the regulation of the UPR is more complicated; at least three mechanistically distinct pathways (Ire1, ATF6, and Perk) operate in parallel to sense unfolded proteins in the ER. Each activates distinct transcription factors that collaborate to trigger a continuum of transcriptional programs in a tissue-specific manner [6]. Among other genes, the ATF6 pathway increases transcription of *XBP1* mRNA [19–23], therefore more of the transcription factor XBP1 is produced upon splicing of its mRNA by Ire1. A similar information network affording “gain control” to the UPR is observed in yeast: the concentration of the *HAC1* mRNA increases 3- to 4-fold when yeast cells are subjected to particularly severe ER stress conditions [24]. This new state, called Super-UPR (S-UPR), allows cells to synthesize more Hac1 protein, yielding a qualitatively different transcriptional output. The up-regulation of the *HAC1* mRNA during S-UPR conditions is necessary for cell survival. The molecular machinery that senses the S-UPR signal and transmits it across the ER membrane is not yet known, but it is clear that it does not require Ire1 [24].

The set of UPR targets includes key players in ERAD [25,26]. ERAD mediates the retro-translocation of unfolded proteins from the ER lumen into the cytosol for degradation by the proteasome. In this way, ERAD complements other UPR targets—such as chaperones and protein-modifying enzymes, whose up-regulation positively facilitates protein folding—by removing hopelessly misfolded proteins from the ER. Proteins entering the ERAD pathway, however, have to traverse the membrane in reverse and presumably do so as an unfolded chain through a protein translocation channel in the membrane. Severely misfolded proteins and protein aggregates might be difficult to unravel and degrade by this mechanism.

An alternative pathway that targets proteins for degradation is autophagy. Autophagy describes a collection of pathways by which sections of the cytoplasm, including its organelles, can become sequestered into membrane-bounded compartments that then fuse with the vacuole (or lysosomes), where their content is degraded by acid hydrolases [27]. In this way, whole organelles can be degraded, regardless of their size or the folding state of their constituent proteins. Many of the components that mediate autophagy have been identified [28–31] and extensively characterized.

Autophagy pathways differ in their selectivity. Macro-autophagy, for example, is induced by starvation and serves to encapsulate and degrade non-selectively large portions of the cytosol [32] and organelles suspended in it, including mitochondria [33] and segments of the ER [34]. This provides cells with badly needed nutrients in the form of metabolites derived from digested proteins and macromolecular structures (auto-cannibalism) [35]. How particular regions of the cytoplasm are chosen to become enclosed in autophagosomes is unknown, as is the origin of the double membrane structure that sequesters them. However, it has been shown that the early secretory pathway contributes to the assembly of autophagosomes [36–38]. By contrast to macro-autophagy, pexophagy and mitophagy are highly selective processes that degrade an excess of peroxisomes and mitochondria, respectively, under growth conditions that change the requirement for these organelles [39,40]. It has been proposed that marker proteins are selectively displayed on no longer needed or damaged organelles, and direct their sequestration. Most of the components that mediate degradative autophagy are also shared by the biosynthetic cytoplasm-to-vacuole targeting (Cvt) pathway [41–43], which operates constitutively to deliver a subset of content proteins to the vacuole during their biosynthesis [44]. The degradative autophagy and biosynthetic Cvt pathways are morphologically and topologically similar and share many components.

Here, we describe an unexpected link between the UPR and autophagy. We show that under UPR-inducing conditions, ER membranes become selectively sequestered in autophagosome-like structures, utilizing components shared with other autophagic processes. We discuss how this ER-selective branch of autophagy, or ER-phagy for short, and the UPR might be physiologically linked during UPR-induced ER proliferation.

## Results

### The ER Expands during Induction of the UPR

**Figure 1:**
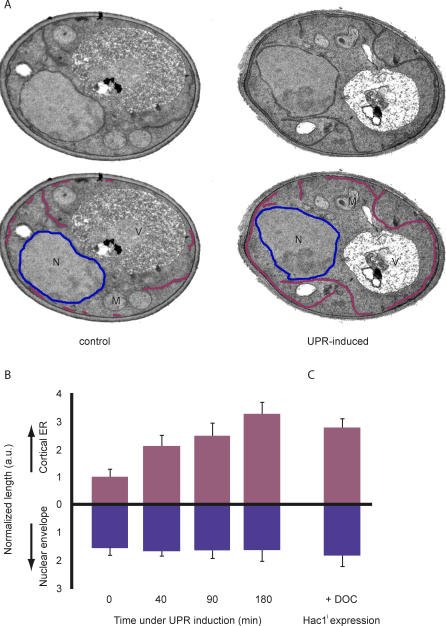
ER Proliferation under UPR-Inducing Conditions (A) Determination of ER abundance in control and UPR-induced cells. Representative cells are shown. The UPR was induced in wild-type cells by addition of DTT. Ultrastructure of control cells and UPR-induced cells was analyzed using ImageJ. The lower images show traces of cortical ER (represented in magenta) and the nuclear envelope (NE, in blue). Vacuoles, nuclei, and mitochondria are indicated as V, N, and M, respectively. (B) Quantification of the ER proliferation during the UPR. UPR was induced and cells were collected for EM at the indicated time points. Length of the ER (as traced in [A]) was measured and divided by the area of the section. Data are plotted relative to time 0. Measurements for each time point correspond to the mean of 25 independent cell images. (C) Expression of *HAC1^i^* was induced by addition of 100 μM DOC for 3 h. ER was quantified as described above in (B).

To ask whether activation of the UPR alters ER structure or abundance, we examined cell thin sections by electron microscopy (EM). To this end, we collected exponentially growing wild-type cells treated with dithiothreitol (DTT) to induce the UPR, and compared them to untreated cells. As shown in Figure 1, the majority of the ER was found at the periphery of the cell (Figure 1A, ER, traced in magenta) or forming the nuclear envelope (Figure 1A, NE, traced in blue).

Even a cursory glance at the images revealed that a massive expansion of the ER occurred after UPR induction. To quantify this effect over time, we measured the cumulative length of the ER in individual EM sections and normalized the results to the area of the cell. As shown in Figure 1B (magenta bars), by this metric, the amount of ER increased more than 3-fold over a 3-h time course after addition of DTT. By contrast, the amount of NE remained constant (Figure 1B, blue bars), indicating that the nuclear volume remained unchanged—thereby serving as a convenient internal control. Proliferation of the ER was rapid, doubling 40 min after the addition of DTT.

To determine whether the observed morphological changes were a direct consequence of the induction of the UPR, we activated the UPR transcriptional program downstream of Ire1 without misfolding proteins in the ER. To this end, we expressed the spliced form of the *HAC1* mRNA (*HAC1^i^* mRNA, for induced) from a regulated glucocorticoid receptor-activated promoter. We induced *HAC1^i^* mRNA in *hac1Δ* cells by addition of deoxycorticosterone (DOC), which binds to the glucocorticoid receptor expressed in these cells and activates it [18]. The amount of ER expansion during *HAC1^i^* mRNA expression was similar to the increase observed during DTT treatment, indicating that activation of the UPR by Hac1 is sufficient to induce the observed ER proliferation (Figure 1C).

**Figure 2:**
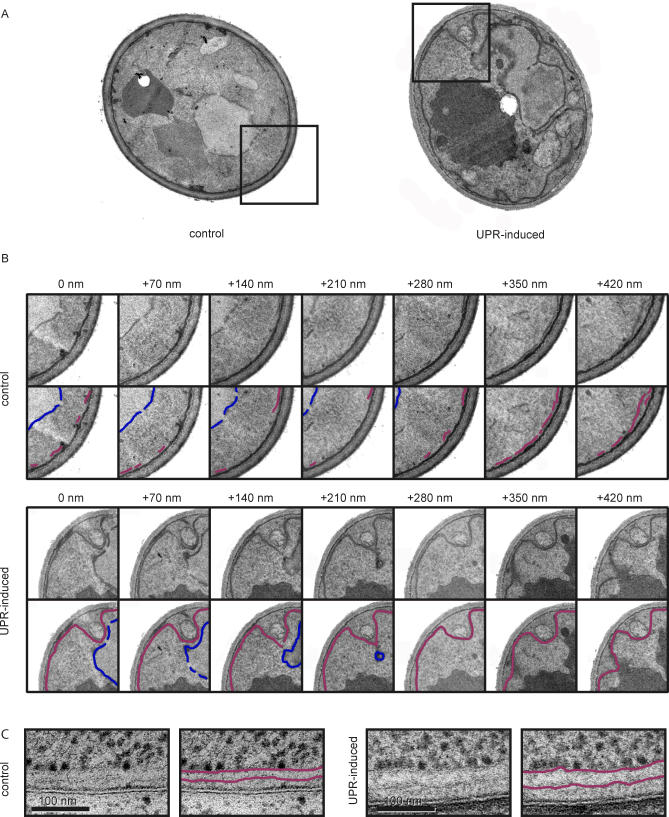
The ER Morphologically Changes during the UPR (A) Control cells and UPR-induced cells were used to analyze and follow the ER within a single cell using EM. Boxes indicate the areas magnified in (B). Cells shown here correspond to the full section of the images labeled “+140 nm” in (B). (B) Serial section of control and UPR-induced cells. Sections are separated by 70 nm on the *z*-axis. ER is represented in magenta and NE in blue. (C) Electro micrographs from control and UPR-induced cells showing that the distance between ER membranes increases during the UPR. For a better preservation of the ultrastructure, samples for this experiment were prepared using high-pressure freezing/freeze substitution techniques (see Material and Methods).

In addition to ER proliferation during the UPR, we observed that the continuity of the ER membrane system increased significantly within a section (Figure 2A). In sequential 70 nm–thick serial sections, short stretches of ER appeared and disappeared in control cells, whereas we could trace a continuous ER over many sections in UPR-induced cells (Figure 2B). This observation suggests a change from predominantly tubules or very small sheets in control cells, to expansive sheets in UPR-induced cells. The expansion of the ER measured in Figure 1B, therefore, is likely an underestimation of both membrane area and organelle volume. Moreover, we observed that the spacing between ER membranes was significantly increased in the expanded UPR-induced ER (Figure 2C; ER membrane distance = 31 ± 5 nm in control cells versus 48 ± 6 nm in UPR-induced cells). We observed this effect qualitatively in fixed permanganate-stained sections, but performed a more accurate distance measurement between ER membranes in flash-frozen/freeze-substituted sections to minimize the chance of specimen distortion [45]. Thus, even without considering the altered geometry of a possible tubule-to-sheet transition, ER volume expands about 5-fold upon UPR induction (3.3-fold expansion of length × 1.5-fold expansion of width).

### Autophagosome-Like Structures Form in a Subset of UPR-Induced Cells

**Figure 3:**
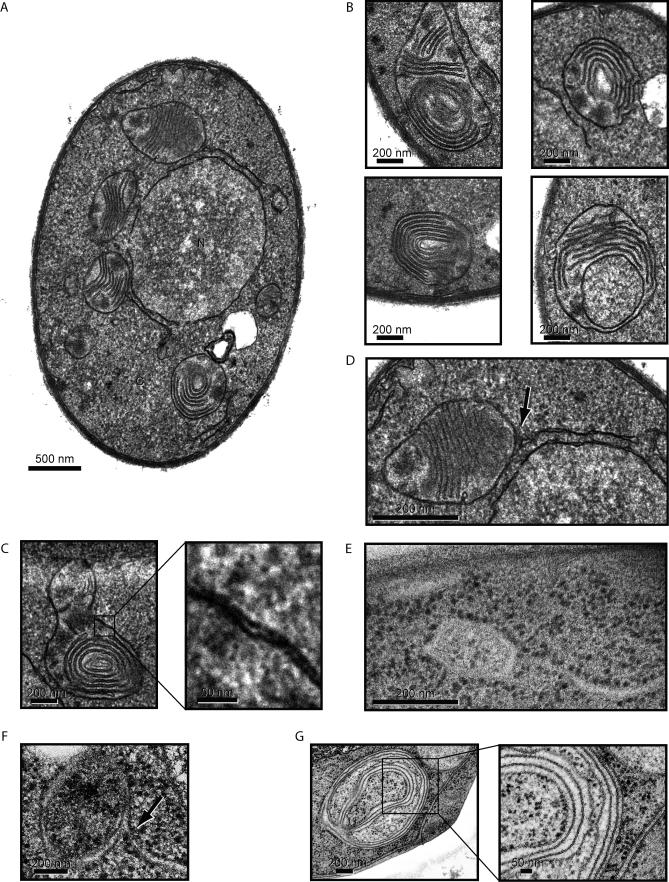
Characterization of ER-Containing Autophagosomes (ERAs) during the UPR (A) Images of representative DTT-treated wild-type cells that contain ERAs. Nuclei and cytoplasm are indicated as N and C, respectively. (B) Enlargement of representative images of ERAs from different cells. The bottom right image is likely to show a section through a cup-shaped ERA. Note that there are no connections between the stacked cisternae and the envelope. (C) High magnification of the ERA double membrane envelope. (D) Some ERAs are found attached to or are in close proximity to ER tubules/sheets (indicated by the arrow). Note that the section in (A) includes two such junctions. (E) High-pressure freezing/freeze substitution image of an ERA linked to an ER tubule/sheet. The osmium/lead staining used in this technique visualizes ribosomes and demonstrates that the outer ERA envelope membrane, but not the stacked internal cisternae, are tightly studded with ribosomes, indicating that they originate from ER membranes. (F) High-pressure freezing/freeze substitution image of an ER-ERA junction using an improved protocol to visualize membranes. (G) Using the same technique as in (F), we visualized the internal membrane content of an ERA. Note that both portions of the internal membranes and of the sequestering double membrane envelope contain bound ribosomes, and hence are likely derived from the ER.

Unexpectedly, we observed that a fraction of UPR-induced cells accumulated large amounts of double membrane–bounded, autophagosome-like structures packed with tightly stacked membrane cisternae (Figure 3A and 3B). We show below that, the content membranes are derived from the ER, and henceforth refer to these structures as ER-containing autophagosomes, or ERAs. ERAs were present in more than 20% of the cells 3 h after the UPR-induction. Significantly, none of the cells in the population containing ERAs had proliferated ER. ERAs show characteristic features of autophagosomes: they are surrounded by a double membrane (Figure 3C) and have similar sizes (300 to 700 nm) [46,47]. Frequently, the delimiting outer membranes connected to tubular or single sheet extensions (Figure 3A and 3D, arrow). To determine if ERAs are derived from the ER, we examined flash-frozen/freeze-substituted sections stained with osmium. In these samples, we found that the outer membrane of ERAs and the extensions were densely studded with ribosomes, suggesting that these membranes are indeed derived from ER (Figure 3E).

The common specimen preparation technique used in Figure 3E does not allow to visualize membranes adequately. While trying to optimize the procedure, we found that inclusion of 3% water during the osmium fixation/substitution step vastly improved membrane visualization in the images, as previously reported [48]. Representative images obtained with this improved technique are shown Figure 3F and 3G, which strongly reinforces the notion that the delimiting membrane of ERAs is continuous with ribosome-studded ER membranes. In the image shown, the continuity of the bilayer can be traced neatly through the junction where the membrane extension meets up with an ERA. Figure 3G show a cross section through an ERA, with clearly visible content of membrane stacks. Note that the sequestered membranes are ribosome-free where they are tightly stacked, but contain membrane-bound ribosomes in regions where they are less tightly apposed.

**Figure 4:**
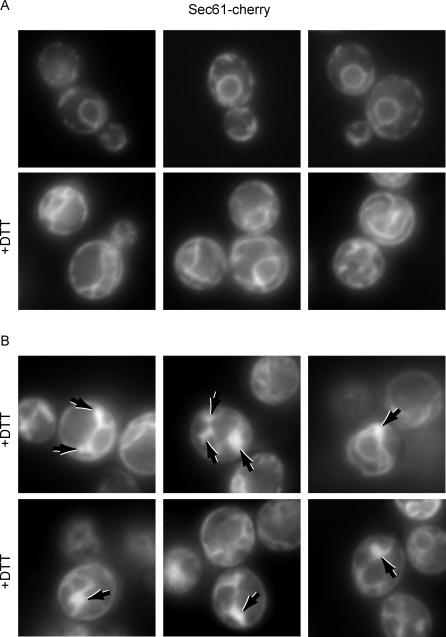
Fluorescence Visualization of an ER Marker after UPR Induction (A) Cells treated with the UPR-inducing drug DTT (+DTT) or with no drug were visualized using a fusion protein between the translocon component Sec61 and the red-fluorescent protein “cherry.” Top panels show untreated cells, and bottom panels show representative UPR-induced cells. (B) Representative images showing UPR-induced cells that contain ERAs (indicated by arrows).

Examination of the ER at a 3-h time point after UPR induction by fluorescent microscopy in cells expressing a Sec61-cherry fusion protein [49] revealed proliferated ER in 80% of the cells (Figure 4A, +DTT, bottom row), in agreement with the EM images shown in Figure 1. By contrast, 20% of the cells showed multiple distinct and intensely fluorescent cytoplasmic bodies (Figure 4B, arrows). Their abundance per cell, their appearance at late (3 h) but not early (90 min) time points after UPR induction, the penetrance of their appearance in 20% of the cells in the population, and their appearance in cells that lack expanded ER are each consistent with the notion that these structures correspond to the ERAs observed by electron microscopy.

**Figure 5:**
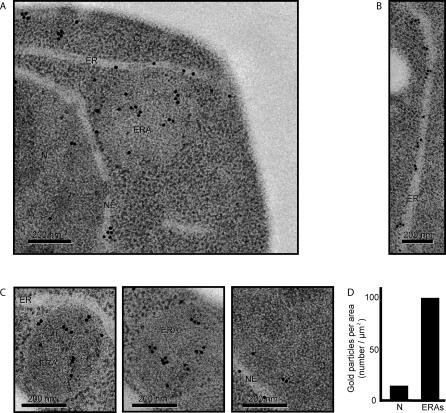
Immunogold Labeling of ERAs with an Antibody Directed against an ER Membrane Marker (A) Representative section of a cell immunolabeled against a myc-tagged Sec63, an integral ER membrane protein. As a primary antibody, we used a rabbit polyclonal anti-myc and, as a secondary, we used 15-nm gold particles–conjugated anti-rabbit antibody. Nucleus, nuclear envelope, ER, and ERA are indicated as N, NE, ER, and ERA, respectively. (B) High magnification of an electron micrograph of a section of ER. Quantification showed that there are 5 ± 2 gold particles per linear micrometer of ER. (C) High magnification of ERAs. To predict how many gold particles one should expect in a particular ERA, we first calculated and averaged the amount of ER (expressed as length in linear micrometers) present in an ERA (similar to the ones shown in Figure 3B), and normalized the value for its area. These calculations determined that there are 20.8 ± 3.3 μm of ER per μm^2^ inside the ERAs. These values allowed us to predict how many gold particles would be expected over a section of an ERA if it were packed with ER membranes. Two representative ERAs are shown. The ERA shown in the middle picture should hold 2.4 μm of ER inside and, therefore, should have 12 gold particles. We counted 12 gold particles. The ERA on the right could contain 2.7 μm of ER and should contain14 gold particles; we counted 16 gold particles. The image on the right shows a representative view of a nucleoplasmic region. (D) Quantification of gold-labeling density per area. To assess the signal-to-noise ratio of our immunogold-labeling procedure, we assessed background labeling by counting the number of gold particles over an areas of nucleoplasm (N) and over ERAs, and normalized the counts to the respective areas.

To obtain further evidence that the membrane stacks observed in ERAs in the EM images are indeed derived from the ER, we prepared EM images for staining with immunogold, using antibodies directed against an epitope tag of an ER resident protein Sec63 (Figure 5). We obtained selective labeling of clearly identifiable ER structures (Figure 5A and 5B), as well as selective labeling of ERAs (Figure 5C). Quantitation of gold particles per area revealed a signal-to-noise ratio of approximately 7:1 when we compared ERA and nucleoplasm (Figure 5D; in cell sections, the nucleoplasm showed the highest density of background staining). In addition, we found that the density of gold particles over ERA regions closely matched the value predicted from the amount of ER membrane packaged in them (Figure 5C). To reach this conclusion, we determined the density of ER membranes in ERAs from EM sections such as shown in Figure 3B (ER length per area) and the density of gold particles along stretches of cytoplasmic ER in immunogold-stained sections such as shown in Figure 5B.

Taken together, the data presented so far suggest that after UPR induction, the ER proliferates significantly. At later time points after induction, some cells in the population reduce their ER back to uninduced levels, and the striking images shown in Figure 3 suggest that this occurs by sequestering ER membranes into ERAs. Interestingly, Hac1^i^ induction, described in Figure 1C, from the DOC-induced reporter construct led to ER proliferation, but by itself was insufficient to induce ERA formation. Since Hac1^i^ is the only known component relaying Ire1 signaling in yeast [50,51], a Hac1- and Ire1-independent second signal must originate from the ER lumen and be required for ERA formation.

**Figure 6:**
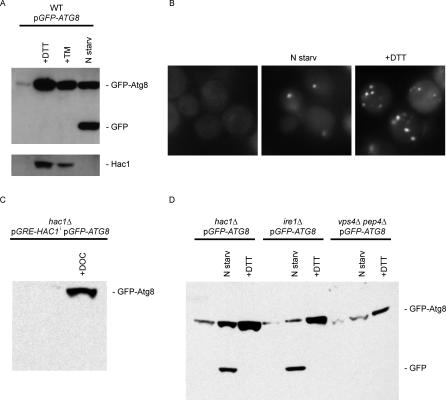
UPR-Induction of the Autophagy Marker GFP-Atg8 (A) Wild-type cells transformed with a plasmid containing GFP-Atg8 were grown for 4 h in synthetic media with no drug, with UPR-inducing conditions (+DTT and +TM), or under nitrogen starvation conditions (N starv), and then harvested for protein preparation. Protein extracts were analyzed by Western blotting using antibodies against GFP (top panel) or Hac1 (bottom panel). Total protein concentration was measured by BCA protein assay. Same concentration of protein was loaded in each lane, and transfer efficiency was checked by Ponceau staining. The identities of the different bands are indicated. (B) Wild-type cells expressing GFP-Atg8 grown under the conditions described above were visualized by fluorescence microscopy. (C) GFP-Atg8 was detected in extracts from untreated *hac1Δ* cells or cells expressing *HAC1^i^* (+DOC) by Western blotting using antibodies against GFP. (D) Western blot using antibodies against GFP of extracts from *hac1Δ, ire1Δ,* or *vps4Δ pep4Δ* cells expressing GFP-Atg8. Mutant cells were grown under regular conditions, UPR-inducing conditions (+DTT), or nitrogen starvation conditions (N starv).

As ERAs structurally resemble autophagosomes, we next sought to determine if there is a functional connection between the UPR-induced ER proliferation and autophagy. To this end, we used Atg8, one of the early mediators of autophagosome formation, as a marker [52,53]. *ATG8* is transcriptionally up-regulated when autophagy is induced, e.g., by nitrogen starvation. Atg8 is a cytosolic protein that becomes lipidated [54,55] and accumulates in pre-autophagosomal structures (PASs) that are in close proximity to the vacuole and can be visualized as dots by fluorescent microscopy in cells expressing green fluorescent protein (GFP)-Atg8 fusion proteins [56–58]. PASs are thought to act as nucleation sites for the formation of autophagosomes, which then fuse with the vacuole where its membranes and internal content are degraded. Because Atg8 (as well as GFP-Atg8) is incorporated into the autophagosomes and subsequently deposited into vacuoles when fusion occurs, GFP-Atg8 has been used as a marker for vacuolar processing: when autophagosomes are delivered to the vacuole, proteolytic cleavage leads to the release of the GFP moiety, which is relatively long lived and hence can be detected as a discrete fragment [59,60]. The data in Figure 6A show that macroautophagy induced by nitrogen starvation leads to a large induction of GFP-Atg8 (compare lanes 1 and 4), about half of which was proteolyzed to GFP at the time point analyzed (Figure 1, lane 4). Proteolysis was no longer observed in *vps4Δ pep4Δ* cells lacking vacuolar proteases (Figure 6D, lane 8). Under nitrogen starvation, no Hac1 was produced (Figure 6A, lower panel), consistent with previous observations that these conditions do not induce the UPR [61].

Similarly, when cells were treated with the UPR-inducing agents DTT or tunicamycin, GFP-Atg8 was strongly induced (Figure 6A). By contrast to Atg8 induction by nitrogen starvation, however, we observed no cleavage of the GFP domain (Figure 6A), even after prolonged incubation of the exponentially growing cells in the presence of the drugs (unpublished data). These surprising results show that the fate of GFP-Atg8—and by inference that of Atg8—is different in UPR-induced and nitrogen-starved cells. When we compared GFP-Atg8 in UPR-induced and nitrogen-starved cells by fluorescence microscopy, we detected a significantly larger number of PASs in UPR-induced cells (Figure 6B).

Expression of *HAC1^i^* mRNA from the glucocorticoid-induced promoter was sufficient to up-regulate GFP-Atg8 (Figure 6C), indicating that DTT and tunicamycin can exert their effects on Atg8 transcription through classical UPR signaling mediated by Ire1 and Hac1. This result was surprising because previous profiling of the total transcriptional scope of the UPR did not identify *ATG8* as a UPR target gene [18]. The paradox is resolved by the data shown in Figure 6D, which demonstrate that, although Hac1^i^ is sufficient to induce Atg8, it is not necessary: Atg8 is strongly induced by DTT and tunicamycin even in *hac1Δ* and *ire1Δ* cells. Our previous study [18] applied stringent filters that required that transcriptional activation of any gene classified as a UPR target gene be Hac1 and Ire1 dependent. *ATG8,* as well as other DTT- and tunicamycin-induced autophagy genes, *ATG5, ATG7,* and *ATG19* [18], were therefore not included in the definition as UPR target genes.

### GFP-Atg8 Localizes in Proximity to ERAs and Facilitates Cell Survival under ER Stress

**Figure 7:**
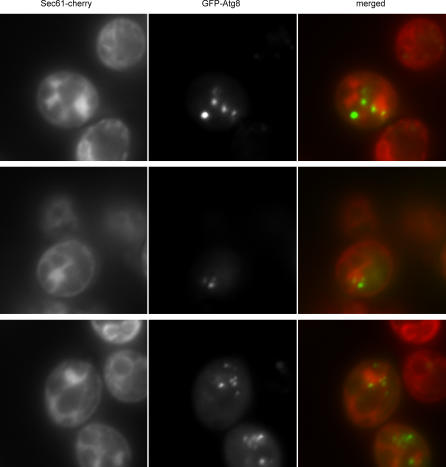
Localization of GFP-Atg8 during UPR Induction Some of the DTT-treated cells shown in Figure 4B expressing GFP-Atg8 and Sec61-cherry (as an ER marker) were visualized using fluorescence microscopy. GFP-Atg8 localizes in close proximity to the ERAs detected by the ER marker.

To determine if ERAs co-localize with GFP-Atg8-staining structures (PASs), we double-labeled cells by co-expressing GFP-Atg8 and Sec61-cherry. Consistent with previous reports [62], we found only a few PASs in uninduced cells (approximately one spot in every 3–4 cells), presumably reflecting a low constitutive rate of autophagy in normally growing cells or the role of PASs in the Cvt pathway. This picture was unchanged at early time points after UPR induction. By contrast, 3 h after UPR-induction, we observed a vast proliferation of PASs (6 ± 2 spots per cell). PASs seemed to be randomly localized in most cells, but upon staining of internal cell membranes with the lipophilic dye FM4–64, were always seen in close juxtaposition to vacuoles or other FM4–64–staining structures (unpublished data), as well as to ERAs in the population of cells that have them (Figure 7). The juxtaposition suggests that PASs may be involved in nucleating ERAs, although they do not co-localize with them. Importantly and in strong support of the notion that Atg8 has a role in ERA formation, we detected no ERAs by EM or by fluorescence microscopy in *atg8Δ* cells.

**Figure 8:**
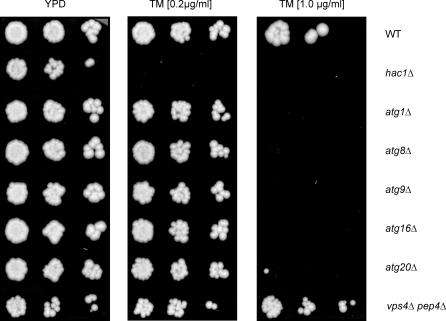
Atg8 and Other *ATG* Genes Are Necessary during UPR Induction Serial dilutions for wild-type, *hac1Δ, atg1Δ, atg8Δ, atg9Δ, atg16Δ,* and *atg20Δ* deletion cells and *vps4Δ pep4Δ* double deletion cells were grown on rich-media plates with no drug (YPD) or with different concentrations of tunicamycin (TM; 0.2 or 1.0 μg/ml). *atg19Δ* gave an identical result to the other autophagy genes shown here (unpublished data).

Given the possible link between autophagy and the UPR, we next asked whether the ability to induce autophagy would give cells a growth advantage under conditions of ER stress. We found that *ATG8* as well as five other autophagy genes tested (*ATG1, ATG9, ATG16, ATG20* (Figure 8), and *ATG19* [unpublished data] [63–66]) are each required for cell growth under strong UPR-inducing conditions: similar to *hac1Δ* cells, *atg8Δ* cells did not grow when plated on media containing 1-mg/ml tunicamycin (Figure 8, right panel). In contrast to *hac1Δ* cells, the autophagy mutants showed no growth defect under less stringent conditions (0.2-mg/ml tunicamycin; Figure 8, middle panel). These results demonstrate a physiologically important relationship between the UPR and autophagy: autophagy augments the UPR to help cells deal with life-threatening consequences of ER stress.

Intriguingly, cell survival under stringent UPR conditions is not dependent on vacuolar proteases: a *vps4Δ pep4Δ* strain showed significant growth even on 1-mg/ml tunicamycin plates (Figure 8, right panel, bottom row). This result is particularly remarkable as this strain is already growth impaired even under normal growth conditions (Figure 8, left panel, bottom row).

## Discussion

The vast scope of the transcriptional profile of UPR target genes previously suggested that the UPR leads to a comprehensive remodeling of the secretory pathway, allowing cells to adjust their ER protein folding and secretory activities according to need. The transcription factor XBP1, the metozoan ortholog of Hac1, was shown in mammalian cells to induce an expansion of the ER [67,68]. Here we show that in yeast, a similar organelle expansion occurs, with the volume of the ER increasing at least 5-fold upon UPR induction. It seems logical for a cell to expand both the machinery and the space dedicated to protein folding to meet the needs of a new physiological state in which proteins stay longer in the ER until they are properly folded or committed to degradation. Proliferating the ER reduces the concentration of unfolded protein, thereby preventing aggregation and giving more time to properly fold proteins or to degrade folding failures. To our surprise, we discovered that an ER-selective UPR-induced form of autophagy, ER-phagy, is activated and is required for cells to survive under conditions of severe ER stress, thus establishing the existence of a physiologically important link between the UPR and autophagy.

Because execution of the UPR transcriptional program leads to ER expansion, it is plausible to assume that ER-phagy serves to provide the opposite effect of reducing the volume of the ER and with it, unfolded ER proteins that have accumulated there. For example, it has been recently shown that the Z variant of human α-1 proteinase inhibitor (A1PiZ) encounters different degradation pathways depending on its expression and aggregation level [69]. Normally, A1PiZ is a substrate of ERAD. However, when A1PiZ is overexpressed, it is sent to the vacuole via the secretory pathway, and any excess of A1PiZ that aggregates inside the ER is targeted to the vacuole via an autophagy pathway, suggesting that ER-phagy may be induced under these conditions. In liver cells, reduction by autophagy of barbiturate-induced expansion of smooth ER was previously observed when the drug was removed [70]; similarly, in UT-1 cells, the expanded ER induced by HMG-CoA reductase (an ER membrane protein) overexpression is reduced by autophagy when the expression of the enzyme is tuned down [71,72]. Thus the UPR may function in conjunction with ER-phagy to balance ER synthesis with ER degradation as part of the homeostatic control network that adjusts ER abundance up and down. Similarly, pexophagy degrades excess peroxisomes when cells switch carbon sources from using fatty acids to other food stuffs [39,73], and mitophagy reduces mitochondrial abundance, e.g., under starvation conditions or under respiring conditions when mitochondria become easily damaged by oxygen radicals [40,74]. For pexophagy, Pex14 has been proposed to have a role in the selective targeting of peroxisomes for degradation [75], but how autophagy targets other organelles for selective sequestration remains an open question.

The ERAD pathway is thought to continually remove unfolded proteins from the ER and channel them to degradation by the proteasome. We have previously shown that ERAD is intimately linked to the UPR; either pathway is necessary for cell survival if the other one is impaired [18,76]. Many ERAD genes are UPR targets, and it was their up-regulation during UPR-inducing conditions that let to the discovery of this connection. By contrast to ERAD genes, autophagy genes were not defined as UPR targets in this study, and the connection between the UPR and autophagy escaped attention. Autophagy genes were excluded from the set of UPR target genes because they are subject to dual control: in response to protein misfolding in the ER, they are induced by Hac1^i^ in the Ire1-dependent UPR pathway, but also by a parallel pathway that can operate in the absence of Ire1 and Hac1. It is likely that this parallel signaling pathway originating from the ER lumen corresponds to the S-UPR previously described to control the expression level of *HAC1* mRNA [24]. Studying the regulation of autophagy genes therefore provides a powerful new experimental angle on deciphering the molecular mechanism of Ire1-independent ER-to-nucleus signaling in yeast. Because Hac1^i^ expression from the glucocorticoid receptor-activated promoter is not sufficient to induce ERA formation, another signal from the ER lumen beyond activating Ire1 must be required. This signal could (directly or indirectly) establish a marker on the ER surface, labeling the organelle as “damaged” for sequestration into ERAs, and it may utilize the same pathway that confers Ire1-independent regulation of *ATG8* transcription and, possibly, of other genes encoding components of the autophagy machinery.

The ERAs observed in this study show several remarkable features. First, they have a strikingly homogenous appearance and are largely filled with tightly stacked membrane cisternae. Second, the Sec61-cherry staining and the Sec63-myc immunogold staining show that the cisternae are derived from the ER. This notion is supported by the observation that cells containing ERAs lack expanded ER, which appears to be consumed during ERA formation. Third, the outer membrane of the delimiting double membrane of ERAs is densely studded with ribosomes and thus also derives—at least in part—from the ER. It has been a longstanding and still unresolved question where the delimiting membrane of conventional starvation-induced autophagosomes comes from [77]. Our finding thus represents a first identification of the origin of the delimiting membrane of an autophagosomal structure by showing that the ER can serve as the membrane source to generate autophagosomal double membranes. Finally, the inner envelope membrane and the membrane of the stacked cisternae for the most part lack bound ribosomes (Figure 3E). The tight packing of the cisternae is consistent with the absence of ribosomes, which could not be accommodated in the approximately 16-nm space between them (a ribosome is approximately 30 nm in diameter). Taken together, these observations suggest that a sophisticated mechanism must exist that peels ER from the cell cortex, strips off most bound ribosomes, compacts the membrane into tight stacks, and packages the stacks selectively and with exclusion of most of the surrounding cytosol into ERAs by enclosing them in an envelope that is also derived—at least in part—from ER membranes. Hence, ERA formation involves a controlled “self-eating” of the ER.

No ERAs are formed in cells lacking Atg8, which is required for early steps in the biogenesis of autophagosomes. We found that during the UPR, Atg8 is first diffusely distributed throughout the cytosol. At later time points, Atg8 coalesces into discrete foci (PASs). This phenomenon occurred in the vast majority of cells (6 ± 2 PASs per cell at 3 h after UPR induction). At the same time point, ERAs formed in 20% of the cells in apparent juxtaposition to PASs. Notably, there is no overlap in staining. Moreover, and in contrast to nitrogen starvation–induced macroautophagy, no Atg8 is delivered to the vacuole (as indicated by the lack of proteolytic cleavage of GFP-Atg8). In principle, two distinct but not mutually exclusive explanations could account for this observation. First, ERA biogenesis selectively excludes co-packaging of Atg8. Although Atg8-containing PASs may nucleate ERA formation, the fluorescence microscopy images show that their localization remains distinct. If a similar process occurred during formation of classical autophagosomes induced by nitrogen starvation, the less-selective sequestrations of surrounding cytosol might non-selectively co-package Atg8 in proximity. Second, ERAs do not fuse with vacuoles when UPR-inducing conditions are maintained. The role of ERAs in the face of ongoing folding stress would therefore primarily be one of sequestration rather than degradation. Consistent with this idea, *vps4Δ pep4Δ* cells lacking vacuolar proteases can live in UPR-inducing conditions despite the fact that they are already sick under normal growth conditions. Cells that are unable to form autophagosomes, however, die upon exposure to folding stress. This is in contrast to macroautophagy during nitrogen starvation, which has the primary purpose to cannibalize portions of the cytoplasm to provide recycled metabolites to the starving cells. *vps4Δ pep4Δ* cells cannot degrade autophagocytosed material and therefore die under these conditions [78]. Either of these two possibilities further supports the notion that ERAs have distinct properties and/or have a distinct fate from classical starvation-induced autophagosomes.

If the main function of ER-phagy is to counteract UPR-induced ER expansion, why do some cells already form ERAs despite ongoing folding stress? We can speculate that an expanded ER could allow cells to isolate potentially toxic unfolded proteins or aggregates into distinct regions of the ER; their preferential packaging into ERAs might serve to make this segregation complete, allow their eventual degradation in bulk, or prevent passing them on to daughter cells. ER-phagy may therefore not only be a homeostatic mechanism to control ER size, but could also serve a detoxification function under certain conditions. The existence of such an additional role of ERAs is supported by the observation that ERAs are not generated in cells expressing Hac1^i^, arguing that ERA formation under UPR-inducing conditions is not triggered by an expanded ER, but requires the actual presence of unfolded proteins. This idea may also explain why ERAs are found only in a fraction of the cells exposed to folding stress. ERA formation under UPR-inducing conditions might only set in when a large load of unfolded proteins has accumulated, and this may be the case only in some cells. UPR activation may induce almost all cells to eventually downsize their ER through ER-phagy, as judged by the widespread generation of extra PASs. However, only some cells may be challenged by unfolded proteins to such an extent that they trigger ER-phagy despite continuing ER stress. The activation of the Ire1-independent arm of the UPR, leading to S-UPR induction, might increase the fraction of cells that form ERAs during folding stress. It will be interesting to determine whether the fraction of cells containing ERAs increases once the folding stress ceases, as the homeostatic function of ER-phagy may then dominate over its detoxification function. In support of such a switch, we have seen in preliminary experiments that ERAs can fuse with vacuoles after UPR-inducing agents have been washed out and the cells recover from stress (S. Bernales and P. Walter, unpublished data). Thus the delivery of ERAs to the vacuole may be a controlled process that can be turned on and off. In summary, many questions about the molecular mechanisms and the cellular functions of ERAs formation remain, but it seems clear that ER-phagy serves as a countermeasure to ER expansion and helps to bring organelle abundance back into balance.

While this work was under review, Yorimitsu et al. [79] independently reported that ER stress triggers autophagy. Their results confirm the transcriptional up-regulation of *ATG8* and GFP-ATG8 foci formation reported here. Moreover, the authors show that ER stress–induced Atg8 is activated by lipid modification, and that the formation of GFP-ATG8 foci depends on *ATG12,* indicating that these structures correspond to PASs seen during starvation-induced macroautophagy. One significant difference is that Yorimitsu et al. report that GFP-Atg8 is degraded, whereas we do not see degradation (Figure 6). This difference is likely due to growth conditions, as they allow cells to go into stationary phase in which starvation-induced macroautophagy is turned on.

After our work was accepted for publication, Ogata et al. [80] reported that autophagy is activated and promotes cell survival upon ER stress in mammalian cells.

## Materials and Methods

### Yeast strains and plasmids.

Strains used in this study were derived from the wild-type strain W303. The *ire1Δ* and *hac1Δ* strains are as described [7,13]. All the *ATG* deletions, the *PEP4/VPS4* double deletion, and the Sec61-cherry strain were derived from the W303 strain by using PCR-based knock-out strategies [49,81]. Strains expressing GFP-Atg8 were transformed with the plasmid pRS316-GFPAtg8p (kindly provided by Yoshinori Ohsumi, National Institute for Basic Biology, Japan). Strains used in Figures 1C and 6C are as previously described [18].

### Cell culture and plates.

Yeast cells were grown in YPD (Figures 1, 2, and 3) or in defined synthetic medium (Figures 4–7) at 30 °C to log phase. For nitrogen starvation experiments, cultures were grown as described [32]. To induce the UPR in liquid medium, cells were treated with 8 mM dithiothreitol (DTT) or 0.2 μg/ml tunicamycin (TM).

Serial dilution experiments (Figure 8) were performed by growing cells at 30 °C to midlog phase. Cells were diluted 5-fold between consecutive positions and then plated on YPD plates, either in the absence or in the presence of 0.2-μg/ml or 1.0-μg/ml TM. Plates were incubated at 37 °C. Induction of Hac1^i^ using the glucocorticoid system was performed as described [18].

### Isolation and detection of protein.

For each condition, total yeast proteins were extracted from 5–10 optical densities (ODs) of exponentially growing cells. To this end, cells were first collected by centrifugation at 5,000 rpm for 5 min. The cell pellets were frozen in liquid nitrogen. The pellets were then resuspended in 200 μl of a solution containing 8 M urea, 50 mM HEPES (pH 7.4), and vortexed with 100 μl of glass beads for 5 min at maximum intensity. Cells extracts were then incubated at 100 °C with 20 μl of 25% SDS. Then, to separate the glass beads from the cell lysate, the bottom of the tube was pierced, placed inside a new 1.5-ml tube, and centrifuged at 1,000 rpm for 30 s. Flow through was collected and centrifuged at maximum speed for 5 min. The supernatant was collected, and protein concentrations were determined by the BCA assay (Bio-Rad Protein Assay, Hercules, California, United States).

For protein detection, 20 μg of total protein were loaded per lane in NuPAGE 10% Bis-Tris Gels (Invitrogen, Carlsbad, California, United States) and separated by electrophoresis. Proteins were then transferred to Protran BA83 nitrocellulose membranes (Whatman Schleicher & Schuell BioScience, Keene, New Hampshire, United States) and analyzed by Western blotting techniques. GFP-Atg8 (Figure 6) was detected using a mouse anti-GFP monoclonal antibody (Molecular Probes, Eugene, Oregon, United States); Hac1 was detected using a polyclonal antibody raised against the carboxy-terminus (Figure 6A) [13].

### EM.

Two different techniques were used to analyze the ultrastructure of cells. First, we used paraformaldehyde fixation followed by KMnO_4_ staining to best visualize membrane structures (Figures 1A, 2A, 2B, 3A, 3B, 3C, and 3E) [82]. To this end, 10 OD units of exponentially growing cells were collected by centrifugation, and the cell pellet was then resuspended in 1 ml of fixative media (1% glutaraldehyde [EMS, Hatfield, Pennsylvania, United States], 0.2% paraformaldehyde [EMS], and 40 mM potassium phosphate [pH 7.0]) for 5 min at room temperature. Cells were then spun down and resuspended in 1 ml of fresh fixative media for 50 min on ice. After the incubation, cells were washed twice with 1 ml of 0.9% NaCl and once with 1 ml of water. Cells were next resuspended in 2% KMnO_4_ for 5 min at room temperature, centrifuged, and resuspended again in fresh 2% KMnO_4_ for 45 min at room temperature. Then we dehydrated the cells by consecutive 15-min washes with graded ethanol (50%, 70%, 80%, 90%, 95%, and 100%). For embedding, we used the Low Viscosity Embedding Media Spurr's Kit (EMS). Cells were infiltrated by 2-h incubations with a 3:1, 1:1, 1:3 dehydrating agent/embedding medium. Then, cells were resuspended in pure embedding medium and incubated at room temperature overnight. The next day, cells were resuspended in fresh embedding medium and cured for 24–48 h at 70 °C.

In addition, we used a high-pressure freezing/freeze substitutions technique known to be less prone to fixation artifacts and dimensional distortions (Figures 2C and 3D). We fixed cells using the Leica EM PACT2 High Pressure Freezer and freeze-substituted them in 2% OsO_4_ plus 0.1% uranyl acetate in the Leica EM AFS2 (Leica, Wetzlar, Germany). Fixed cells were then washed three times with pure acetone and embedded as described above. In the images shown in Figure 3F and 3G, the OsO_4_/uranyl acetate freeze-substitution solution contained 3% water [48]. For the immunogold labeling, we freeze-substituted the samples in 0.1% glutaraldehyde, 0.25% uranyl acetate, and 0.01% OsO_4_, and we embedded them using the LR white resin system [45].

Blocks from these preparations were next sectioned and post-stained with 2% uranyl acetate in 50% methanol for 5 min and Reynold's lead citrate for 2 min. The final material was visualized on a FEI Tecnai 20 electron microscope (FEI, Hillsboro, Oregon, United States). Images were processed and analyzed using ImageJ (W. S. Rasband: http://rsb.info.nih.gov/ij/).

### Light microscopy.

To analyze cells by fluorescence microscopy, we first treated microscope cover glasses with concanavalin A for 30 min. We then deposited 10 to 20 μl of cell culture on a microscope slide and covered it with the treated cover glass. Prepared cells were visualized on a Zeiss Axiovert 200M fluorescence microscope (Zeiss), and images were processed using ImageJ.

## Abbreviations

A1PiZ: Z variant of human α-1 proteinase inhibitor
Cvt: cytoplasm-to-vacuole targeting
EM: electron microscopy
ER: endoplasmic reticulum
ERA: ER-containing autophagosome
ERAD: ER-associated protein degradation
ER-phagy: ER-selective branch of autophagy
GFP: green fluorescent protein
NE: nuclear envelope
PAS: pre-autophagosomal structure
S-UPR: Super-unfolded protein response
UPR: unfolded protein response
